# Association study of two inflammation-related polymorphisms with susceptibility to hepatocellular carcinoma: a meta-analysis

**DOI:** 10.1186/s12881-014-0092-7

**Published:** 2014-08-10

**Authors:** Jiajing Liu, Bo Xie, Shuilian Chen, Feng Jiang, Wei Meng

**Affiliations:** 1 Department of Epidemiology, Key Laboratory of Public Health Security, Ministry of Education, School of Public Health, Fudan University, Shanghai 200032, China; 2 Department of Human Resources, Shanghai East Hospital, Tongji University, Shanghai, China; 3 Department of Medical Insect Vectors Control, Center for Disease Control and Prevention, Pudong New Area, Shanghai, China

## Abstract

**Background:**

Inflammation is a response of body tissues to injury or irritation. Small RNAs, such as miR-146a and miR-499, participate in various processes of tumorigenesis. A recent study indicates that inflammation and abnormal immune responses may promote malignant progression in cancer development, indicating that inflammation-related polymorphisms such as miR-146a rs2910164 and miR-499 rs3746444 are crucial. However, studies on the association of these two polymorphisms with hepatocellular carcinoma (HCC) are inconclusive and inconsistent. We aimed at accessing the combined result of reported studies and make a more precise estimate of the relationship.

**Methods:**

Meta-analysis was performed on the associations between the miR-146a rs2910164 C > G and miR-499 rs3746444 T > C polymorphisms and hepatocellular carcinoma, using: allele contrast, dominant, and recessive models. A total of 12 studies(8 on miR-146a rs2910164 and 4 on miR-499 rs3746444) with three populations (Chinese, Korean, Turkish) were included in this study.

**Results:**

Results show that both allele frequency and genotype distributions of miR-146a rs2910164 polymorphism are significantly associated with susceptibility to HCC (G versus C: OR = 1.153, 95% CI 1.083–1.228, *P* < 0.001; GC versus CC: OR = 1.165, 95% CI 1.054–1.286, *P* = 0.003; GG versus CC: OR = 1.361, 95% CI 1.192–1.553, *P* < 0.001; GG/GC versus CC: OR = 1.213, 95% CI 1.104–1.333, *P* < 0.001; GG versus GC/CC: OR = 1.210, 95% CI 1.080–1.356, *P* < 0.001). Our data suggest that people with G allele have a higher susceptibility to HCC as compared to those with C allele. However, meta-analysis failed to detect associations between miR-499 rs3746444 and HCC risk under each genetic model tested. Subgroup analysis showed that Chinese population with CC genotype are more vulnerable to HCC (OR = 2.171, 95% CI = 1.149–4.104, P = 0.017) than those with TT genotype.

**Conclusions:**

We conclude that rs2910164 in miR-146a may confer susceptibility to HCC, especially in the Chinese population. No significant association was found between miR-499 rs3746444 and HCC, but subgroup study showed that subjects with CC genotype are more vulnerable to HCC than TT genotype in the Chinese population.

## Background

Hepatocellular carcinoma (HCC) is one of the most common malignant tumors and the third most common cause of cancer-related mortality worldwide [1], especially in China. The incidence and mortality rates of this debilitating illness are almost equal due to associated poor prognosis and high fatality. About 695,900 people die each year in China from primary carcinoma of the liver, which contributes to almost 45% mortality globally [2]. Research on the mechanism of cancer, ongoing for over a century, has so far revealed that infections such as Hepatitis B and C are major causes of HCC, and environmental factors such as excessive alcohol intake, cigarette smoking, and obesity are factors that may increase the risk of HCC [3]. However, a recent study showed that only a fraction of HBV-infected patients developed HCC during their lifetime [4], thus implying that genetic factors may also contribute to carcinogenesis.

Since microRNAs (miRNAs) were first described in 1993, these small, evolutionarily conserved, endogenous, single-stranded, non-coding RNA molecules [5], typically 20 to 22 nucleotides in length, have been found to post-transcriptionally function as negative regulators of gene expression and function [6]. Growing evidence suggests that miRNAs regulate a wide range of biological processes, including development, cell differentiation, inflammation, proliferation, and apoptosis [7], and they even play a crucial role in initiating human cancers [8]. Therefore, dysregulated miRNAs, which otherwise function as suppressors of tumors or ontogenesis, may be responsible for the initiation, progression, and treatment outcomes of various forms of cancer [9]. Single nucleotide polymorphisms (SNPs) are the most common type of genetic variations associated with population diversity, disease susceptibility, and individual response of medicine [10].

Recent studies have demonstrated that inflammation and abnormal immune responses play an important role in HCC development. Kazuo Tarao *et al*. concluded that inflammation in the background non-cancerous cirrhotic portion of the liver could evoke malignant progression in HCC development [11]. Nonsteroidal anti-inflammatory drugs (NSAIDs) have also been shown to reduce chronic inflammation and the risk of various forms of cancer [12]. Inflammation related to polymorphism may alter immune response and thus affect the development of HCC.

miR-146a rs2910164 is located in the stem region opposite the mature miR-146a sequence. This C > G polymorphism results in a change from C:U pair to G:U mismatch in the stem structure of miR-146a precursor, which may have an impact on the development of HCC [13]. miR-499 rs3746444 is located in the stem region opposite the mature miR-499 and results in a change from T:U pair to C:U mismatch in the stem structure of miR-499 in the miR-499 gene [14]. miR-499 has been recognized as an ideal biomarker for carcinogenesis due to its involvement in several biological processes, such as cellular senescence, apoptosis, inflammation, and immune response, all of which are crucial in the development and progression of cancer [14]–[16].

To date, several groups have reported polymorphisms such as rs2910164 in miR-146a and miR-499 rs3746444, which could be biomarkers of susceptibility to HCC [17]; however, the associations remain controversial and inconclusive due to the relatively small sample size that was analyzed. For rs2910164 in miR-146a, Xu *et al*. [18] reported that the polymorphism was significantly associated with the risk of HCC development under a dominant model in the Chinese population, while Zhou *et al*. [19] suggested a lack of association between the SNP and primary liver cancer risk. The lack of power to detect small effects of gene polymorphism on cancer is a major concern. For miR-499 rs3746444, Kim *et al*. [20] failed to identify the association between the SNP and HCC, while Zhou *et al*. [19] reported that the polymorphism was strongly related to HCC risk. Reports of meta-analysis from different groups as well show contradicting opinions on this matter [4],[21]–[23]. Hence, we performed a meta-analysis of all the eligible studies, hoping to provide more clarity by systematically summarizing the existing data.

## Methods

### Identification of eligible studies

We carried out a computer-based search of the most commonly used database, including PubMed, EMBASE, Web of Science, Cochrane Central Register of Controlled Trials, China National Knowledge Infrastructure (CNKI),China Biological Medicine Database (CBMD) and VIP(Chinese) to identify all studies examining the relationship between miR-146a rs2910164, miR-499 rs3746444and HCC. The following keywords and subject terms were used for searching: “miR-146a”, “microRNA”, “rs2910164”, “miR-499”, “rs3746444”, “polymorphism”, “Hepatocellular Carcinoma”, “liver cancer”, “HCC”,” Liver neoplasm” as well as combinations, without any restriction on publication date and language by two independent investigators (Jiajing Liu and Bo Xie) up to February, 2013. References of retrieved articles were also scanned. Corresponding authors of published papers without sufficient data were contacted by e-mail for more information. The design of the meta-analysis meets the requiries of moose checklist (Additional file 1).

### Criteria for study selection

In order to meet the aim of the meta-analysis, strict criteria were used to identify the relevant published studies:

(i) the study followed a case–control design and the participants were in both groups voluntarily joined this study with informed consents; (ii) the study was designed to evaluate the associations between the SNP miR-146a rs2910164 or miR-499 rs3746444 polymorphisms and susceptibility to HCC; (iii) participants recruited from comparable populations with similar demographic background in control and case groups; (iv) a proper diagnosis criteria of HCC to determine the disease status respectively; (V) detailed genotype data were provided for the calculation of odds ratio (OR) and 95% confidence interval (95%CI); (vi) proper method to determine the genotypes with detailed description of method used; (vii) Hardy-Weinberg equilibrium (HWE) reached.

**Figure 1:**
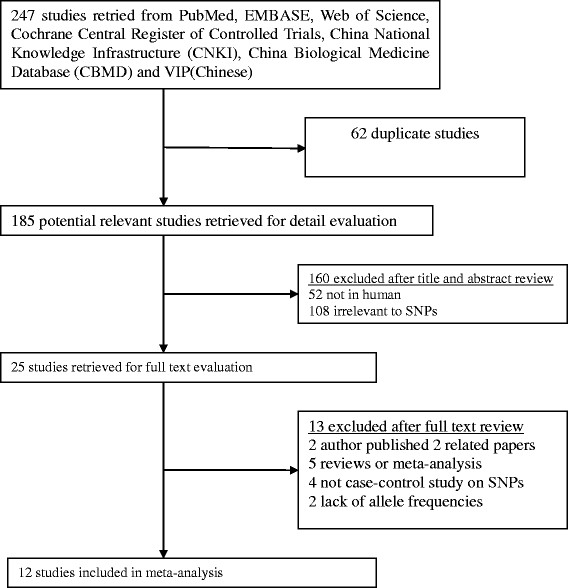
Flow diagram of study identification.

We excluded the following: (1) studies that contained overlapping data, the latest study was included; (2) studies in which the number of wild genotypes could be ascertained; (3) the manuscript was published as a review or abstract, not a full original paper. The details are demonstrated in Figure 1.

### Statistical analysis

The Hard-Weinberg Equilibrium (HWE) was evaluated by the goodness-of-fit chi-square test for the control groups of each study (significance set at *P* = 0.05, *P* < 0.05 was considered a departure from HWE). If the control groups were not in HWE, sensitivity analysis or subgroup analysis was performed to test the robustness of the findings.

The odds ratio (OR) corresponding to the 95% confidence interval (95% CI) was calculated to assess the strength of association between rs2910164 and rs3746444 polymorphisms and susceptibility to HCC. We performed a meta-analyses using allele frequency comparison; additive model; dominant model; and recessive model. The significance of pooled ORs was determined by *Z*-test and *P* < 0.05 was considered statistically significant. Heterogeneity assumption was checked by the chi-square-based *Q*-test, and a *P* > 0.05 indicates a lack of heterogeneity among studies [24], so the fixed-effects model (the Mantel-Haenszel method) [25] was applied. Random-effects model (the DerSimonian and Laird method) [26] are more appropriate when heterogeneity is present. Sensitivity analysis was carried out by deleting one single study each time to examine the influence of individual data set on the pooled ORs. Modified Egger's linear regression test and funnel plots are used to detect publication bias. An asymmetric plot suggests a possible publication bias and the *P* <0.05 of Egger’s test was considered representative of statistically significant publication bias [27]. All analyses were performed by using the software Stata version 11.0 (Stata Corp LP, College Station, TX, USA). All reported probabilities (*P* values) were two-sided.

## Results

### Description of included studies

A total of 237 articles were identified, based on the above search criteria, in PubMed, EMBASE, Web of Science, Cochrane Central Register of Controlled Trials, China National Knowledge Infrastructure (CNKI), China Biological Medicine Database (CBMD), and VIP (Chinese). For polymorphism of miR-146a rs2910164, only 8 case–control studies qualified for our meta-analysis [18]–[20],[28]–[32]. In the study by Wang *et al*. [29], the authors stratified their data using three independent subpopulation samples retrieved from three different provinces and analyzed and presented them separately. Therefore, we extracted the data separately and numbered them as Wang-1, Wang-2, and Wang-3. For the polymorphism of miR-499 rs3746444, only 4 studies were included in our meta-analysis. Overall, we identified 8 articles, including 12 samples with 3747 patients and 4779 controls for miR-146a rs2910164 and 4 articles with 746 patients and 1006 controls for miR-499 rs3746444 to evaluate the association of these polymorphisms with HCC in Chinese populations. The characteristics of included studies are summarized in Tables 1 and 2. All the polymorphisms in the controls of these studies were found to occur in frequencies consistent with that of HWE (Tables 1 and 2).

**Table 1:** Characteristics of studies included in the meta-analysis

**Author**	**Year**	**Population**	**Sample size (case/control)**	**Genotyping method**	**Case**			**Control**			**х**^**2**^	**HWE (P value)**
					**GG**	**CG**	**CC**	**GG**	**CG**	**CC**		
Zhou	2011	China	669(186/483)	PCR-RFLP	33	86	67	71	254	158	3.658195	0.055794
Yu	2012	China	200(100/100)	PCR-RFLP	27	45	28	21	46	33	0.443003	0.505677
Xu	2008	China	983(479/504)	PCR-RFLP	80	241	158	58	249	197	2.430179	0.119019
Wang-1	2011	China(Jiangsu)	1722(640/1082)	MASSARRAY	122	330	188	166	551	365	3.171700	0.074924
Wang-2	2011	China(Henan)	583(199/384)	MASSARRAY	38	103	58	61	185	138	0.005863	0.938967
Wang-3	2011	China(Shanghai)	680(277/403)	MASSARRAY	52	128	97	45	188	170	0.420956	0.51646
Li	2012	China	1120(560/560)	AS-PCR	124	302	134	92	288	180	1.670302	0.196218
Zhang	2011	China	1765(925/840)	PIRA-PCR	156	450	319	151	386	303	2.086630	0.148594
Kim	2012	Korean	360(159/201)	PCR-RFLP	14	88	57	24	103	74	1.71898	0.189825
Akkiz	2011	Turkey	444(222/222)	PCR-RFLP	137	75	10	144	67	11	0.758665	0.383747

For miR-146a rs2910164.

**Table 2:** Characteristics of studies included in the meta-analysis

**Author**	**Year**	**Population**	**Sample size (case/control)**	**Genotyping method**	**Case**			**Control**			**х**^**2**^	**HWE (P value)**
					**CC**	**CT**	**TT**	**CC**	**CT**	**TT**		
Kim	2012	Korean	399(198/201)	PCR-RFLP	3	86	109	7	74	120	1.178699	0.277621
Yu	2012	China	205(105/100)	PCR-RFLP	24	45	36	10	36	54	1.147959	0.283977
Zhou	2011	China	716(233/483)	PCR-RFLP	4	88	141	12	100	371	2.701215	0.100272
Akkiz	2011	Turkey	432(210/222)	PCR-RFLP	90	75	45	82	93	47	4.401451	0.055908

For miR-499 rs3746444.

### Meta-analysis of the miR-146a rs2910164 polymorphism and HCC risk

**Figure 2:**
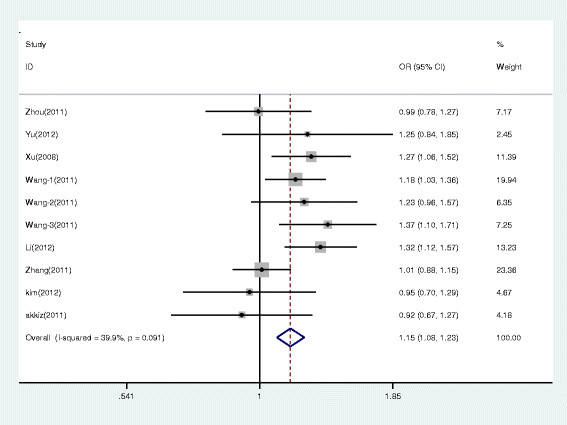
Forest plots under additive model of study between the miR-146a rs2910164 polymorphism and HCC risk.

The results of meta-analysis of the miR-146a rs2910164 polymorphism and HCC risk are shown in Table 3. The meta-analysis was based on 10 independent population samples with 3747 patients and 4779 controls. Q-test in all the models showed no significant heterogeneity. Therefore, all pooled ORs were calculated using fixed-effects model. Significant association was identified between miR-146a rs2910164 polymorphism and susceptibility to HCC in case of all the genetic models (G versus C: OR = 1.153, 95% CI 1.083–1.228, *P* < 0.001; GC versus CC: OR = 1.165, 95% CI 1.054–1.286, *P* = 0.003; GG versus CC: OR = 1.361, 95% CI 1.192–1.553, *P* < 0.001; GG/GC versus CC: OR = 1.213, 95% CI 1.104–1.333, *P* < 0.001; GG versus GC/CC: OR = 1.210, 95% CI 1.080–1.356, *P* < 0.001). The forest plots under additive model of study between the miR-146a rs2910164 polymorphisms and HCC risk are presented in Figure 2.

**Table 3:** Meta-analysis of the miR-146a rs2910164 polymorphisms and HCC risk

**Genetic model**	**Test of association**			**Test of heterogeneity**			**Publication bias (P value)**
	**OR**	**95% CI**	**P value**	**Model**	**I**^**2**^	**P value**	
G v C	1.153	1.083-1.228	<0.001	F	39.9%	0.091	0.816
GC v CC	1.165	1.054-1.286	0.003	F	0.0%	0.690	0.874
GG v CC	1.361	1.192-1.553	<0.001	F	43.2%	0.070	0.990
GG/GC v CC	1.213	1.104-1.333	<0.001	F	6.7%	0.380	0.927
GG v GC/CC	1.210	1.080 -1.356	0.001	F	46.1%	0.054	0.846

(F: Fixed-effects model).

### Subgroup analysis for miR-146a rs2910164

In addition, a subgroup analysis stratified by study characteristics like population (Chinese and other), genotyping method (PCR-RFLP and other), sample size, and significant association was performed for a better understanding of the relationship between the SNP and HCC (Table 4). The study shows a consistently strong association under additive genetic model, except for other populations (OR = 0.939, 95% CI 0.752–1.172, *P* = 0.577) and PCR-RFLP method (OR = 1.103 95% CI 0.982–1.239, *P* = 0.097) subgroups. Though not statistically significant, the PCR-RFLP method still identified G allele as a risk factor (OR > 1) in comparison with C allele. However, other genotyping methods (OR = 1.174, 95% CI = 1.090-1.265, *P* < 0.001), such as MassArray, still found an association between SNP and HCC.

**Table 4:** Subgroup-analysis of the miR-146a rs2910164 polymorphisms and HCC risk

**Subgroup**	**Comparisons**	**Test of association**			**Test of heterogeneity**		
		**OR**	**95%CI**	**P value**	**Model**	**I**^**2**^	**P value**
**Population**							
Chinese	8	1.174	1.099-1.253	<0.001	F	38.5%	0.123
Other	2	0.939	0.752-1.172	0.577	F	0.0%	0.875
**Genotyping method**							
PCR-RFLP	5	1.103	0.982-1.239	0.097	F	25.8%	0.249
Other	5	1.174	1.090-1.265	<0.001	F	54.6%	0.066
**Sample size**							
<700	6	1.129	1.010-1.262	0.033	F	31.3%	0.201
>700	4	1.164	1.079-1.256	<0.001	F	60.0%	0.057

### Meta-analysis of the miR-499 rs3746444 polymorphism and HCC risk

The results of meta-analysis based on 4 independent population samples with 746 patients and 1006 controls for the miR-499 rs3746444 polymorphism and HCC risk are shown in Table 5. Q-test in three models (C versus T, CC versus TT, CC/CT versus TT) showed significant heterogeneity. Therefore, these three pooled ORs were calculated using random-effects model. There was no significant association between HCC and the miR-499 rs3746444 polymorphism under all the genetic models tested (C versus T: OR = 1.118 95% CI 0.773–1.616, *P* = 0.554; CT versus TT: OR = 0.994, 95% CI 0.784–1.262, *P* = 0.963; CC versus TT: OR = 1.266, 95% CI 0.596–2.687, *P* = 0.540; CC/CT versus TT: OR = 1.091 95% CI 0.726–1.641, *P* = 0.674; CC versus CT/TT: OR = 1.265 95% CI 0.924–1.732, *P* = 0.143).

**Table 5:** Meta-analysis of the miR-499 rs3746444 polymorphisms and HCC risk

**Genetic model**	**Test of association**			**Test of heterogeneity**			**Publication bias (P value)**
	**OR**	**95%CI**	**P value**	**Model**	**I**^**2**^	**P value**	
C v T	1.118	0.773-1.616	0.554	R	77.6%	0.004	0.33
CT v TT	0.994	0.784-1.262	0.963	F	42.5%	0.156	0.382
CC v TT	1.266	0.596-2.687	0.540	R	63.8%	0.040	0.827
CC/CT v TT	1.091	0.726-1.641	0.674	R	68.2%	0.024	0.292
CC v CT/TT	1.265	0.924 -1.732	0.143	F	50.8%	0.107	0.884

(F: Fixed-effects model, R: Random-effects model).

### Subgroup analysis for miR-499 rs3746444

Furthermore, we conducted subgroup analysis including those on population (Chinese and other) and sample size (<300 and >300), to analyze characteristic homogeneous groups in all the genetic models and assess heterogeneity among the studies (Table 6). The subgroup analysis under the CC versus TT model showed significant association between miR-499 rs3746444 polymorphism and HCC risk (OR = 2.171, 95% CI 1.149-4.104, *P* = 0.017) in the Chinese population. Subgroup analysis did not show significant heterogeneity under allele frequency contrast model, except in the Chinese subgroup. Although meta-regression analysis failed to identify the source of homogeneity due to the limited number of studies, the subgroup analysis indicated population and sample size as the two major concerns for the source of heterogeneity.

**Table 6:** Subgroup-analysis of the miR-499 rs3746444 polymorphisms and HCC risk

**Subgroup**	**Comparisons**	**Test of association**			**Test of heterogeneity**		
		**OR**	**95% CI**	**P value**	**Model**	**I**^**2**^	**P value**
**C versus T**							
**Population**		OR	95% CI	P value	Model	I^2^	P value
Chinese	2	1.424	0.730-2.776	0.300	R	83.3%	0.015
Other	2	0.949	0.764-1.179	0.636	F	69.9%	0.068
**Sample size**							
<300	1	2.020	1.333-3.063	0.001	NA	NA	NA
>300	3	0.968	0.804-1.165	0.733	F	41.8%	0.179
**CC versus TT**							
**Population**		OR	95% CI	P value	Model	I^2^	P value
Chinese	2	2.171	1.149-4.104	0.017	F	73.4%	0.053
Other	2	1.021	0.638-1.634	0.931	F	29.0%	0.235
**Sample size**							
<300	1	3.600	1.539-8.421	0.003	NA	NA	NA
>300	3	0.998	0.647-1.541	0.994	F	41.8%	0.179
**CC/CT versus TT**							
**Population**		OR	95% CI	P value	Model	I^2^	P value
Chinese	2	1.325	0.962-1.824	0.085	F	73.1%	0.054
Other	2	0.838	0.611-1.148	0.271	F	46.2%	0.173
**Sample size**							
<300	1	2.087	1.184-3.679	0.011	NA	NA	NA
>300	3	0.916	0.716-1.174	0.489	F	24.8%	0.264

### Publication bias

We used modified Egger’s linear regression test to assess publication bias of both, the polymorphisms and HCC risk, under all the genetic models. Symmetrical funnel plots were obtained in the SNP tested in all the models. The results showed that no statistically significant small study or publication bias was present (all *P* values for bias of >0.05). The result confirmed the absence of publication bias in this meta-analysis (Tables 3 and 5).

### Sensitivity analysis

In order to assess the robustness of the meta-analysis results, we carried out a sensitivity analysis. Studies showed a significant effect on the pooled OR, excluded to prevent abnormal results or data causing a deviation from the real association. A single study was deleted each time to examine the influence of the individual data set to the pooled ORs.

For rs2910164, not a single study showed significant effect on the pooled OR. Two independent studies [28],[30] were the main origins of heterogeneity in our meta-analysis. After excluding those studies, strong association between miR-146a rs2910164 polymorphisms and HCC risk was still detected in all the models. The sensitivity analysis indicated that each individual data had no significant influence on the pooled OR, which suggests that the result of our meta-analysis is relatively accurate and robust.

As for rs3746444, one study [19] changed the inter-study heterogeneity in each model comparison. The subgroup analysis earlier showed that population is a significant source of heterogeneity among studies. After the deletion of this study, the heterogeneity vanished, while the association remained insignificant (Table 7).

**Table 7:** Sensitive-analysis of the miR-499 rs3746444 polymorphisms and HCC risk

**Genetic model**	**Test of association**			**Test of heterogeneity**			**Publication bias (P value)**
	**OR**	**95% CI**	**P value**	**Model**	**I**^**2**^	**P value**	
C v T	0.968	0.804-1.165	0.733	F	41.8%	0.179	0.414
CT v TT	0.907	0.699-1.176	0.462	F	2.9%	0.357	0.868
CC v TT	0.998	0.647-1.541	0.994	F	0.0%	0.479	0.266
CC/CT v TT	0.916	0.716-1.174	0.489	F	24.8%	0.264	0.836
CC v CT/TT	1.070	0.756-1.514	0.704	F	0.0%	0.107	0.216

(F: Fixed-effects model).

## Discussion

In general, single nucleotide polymorphism (SNP) variation in miRNA genes has been considered uncommon owing to high evolutionary conservation across species [23]. These small, noncoding RNAs, such as miR-146 and miR-499, have been shown to participate in various processes in tumorigenesis, such as inflammation, cell cycle regulation, differentiation, apoptosis, and invasion [33]. Any minor variation in miRNAs is amplified by hundreds of target genes, and this contributes considerably to the individual’s susceptibility to cancer [6].

In the meta-analysis of the relationship of rs2910164 polymorphism to HCC risk, strong significant association can be found across all the genetic models, which was not in accordance with the previous meta-analysis reported by Wang *et al*. [23]. Unfortunately, results were controversial across the meta-analyses, which may be at least partly due to the fact that Wang *et al*. [23] failed to include all the available studies concerning the association between SNP polymorphism and susceptibility to HCC. In the subgroup analysis, other genotyping method, such as MassARRAY, helped find an association between SNP and HCC (OR = 1.174, 95% CI 1.090–1.265, *P* < 0.001). Additionally, PCR-RFLP method (OR = 1.103, 95% CI 0.982–1.239, *P* = 0.097) shows no significant association in the additive model. This may be a result of many factors. Firstly, the other genotyping method, namely MassArray, is a relatively new genotyping method as compared to PCR-RFLP. It has many advantages such as complete multiple sites in a large sample size. This relatively updated method may cause differential results. The traditional method, PCR-RFLP, is a low-cost, golden standard for genotyping, but cannot accommodate a large sample size. Secondly, MassARRAY has a relatively high false-positive rate, which could affect the significance of the association. Thirdly, the studies with MassARRAY tend to have a larger sample size, which may make up for the lack of power to detect a possible small effect of polymorphism on cancer in small samples of studies. Summarily, this may be the result of multiple factors. The different results in other population may due to the allele frequency difference of this SNP between Chinese and other populations. The G allele frequency in Chinese and Korean populations was 0.535 (Hap- Map-HCB Genome browser release #28) and 0.354 (Hap Map-CHB Genome browser release #28), respectively, while in the Turkish population, it was 0.800 (Akkiz *et al*. [31] control data instead, unable to identify relative data on International Hap Map Project). The significant difference in this comparison supports the finding that population is a major source of heterogeneity.

The functional C > G SNP rs2910164 in the seed sequence of pre-miRNA-146a resulted in mis-pairing of hairpin structures [34]. The C allele gene displayed decreased production of mature miR-146a, as compared to the G allele. The mature miR-146a failed to inhibit target genes, including IL-1 receptor-associated kinase 1 (IRAK1), TNF receptor-associated factor 6 (TRAF6), and papillary thyroid carcinoma 1 gene (PTC1) [34]. This resulted in constant activation by the Toll-like receptors and cytokine receptors and an enhanced inflammatory response [35]. In general, chronic inflammation is associated with persistent cell damage and consecutive regeneration, potentially leading to changes such as fibrosis and cirrhosis and eventually HCC [36].

However, we failed to identify any association between miR-499 rs3746444 polymorphism and HCC risk with all genetic models tested, which was consistent with the results of previous studies [37]. However, strong heterogenicity was detected under most genetic models. The subgroup analysis showed that subjects with CC genotype in the Chinese population are more vulnerable to HCC (OR = 2.171, 95% CI 1.149–4.104, *P* = 0.017) than those with the TT genotype. This result may indicate that CC genotype of miR-499 rs3746444 is a risk genotype and has a higher susceptibility to HCC than any other genotypes in the Chinese population. The optimal free energy change from −62.30 kcal/mol for T to −61.90 kcal/mol for C alleles, suggesting a less-stable secondary structure for the C allele as compared to the T allele [14]. It has been observed that genetic variation in mature miRNA regions could change the conformation of the secondary structure and thereby directly affect both, binding to target mRNAs and the miRNA maturation process [38],[39]. Although our study failed to identify the association between miR-499 rs3746444 polymorphism and HCC, previous reports show an evidence of miRNA contribution to the development of HCC. Lafferty-Whyte *et al*. [15] reported that miR-499 has the potential to regulate processes involved in the induction of cellular senescence, including telomere shortening, oxidative stress, oncogene expression, and signaling of DNA damage. In the sensitivity analysis, one study [19] was identified as a source of heterogeneity and was excluded from our meta-analysis. As the heterogeneity was greatly reduced, the association remained negative with all the genetic models.

In summary, the two miRNAs related to inflammation that were analyzed in this study may have an important influence on HCC. Our results suggest that malignant progression of HCC may be prevented by anti-inflammatory therapy. Additionally, miRNAs play a major role in the pathways they regulate, which should be explored further with respect to autoimmune disease.

### Comparisons with other meta-analysis

Although previous meta-analyses have already reviewed the potential association that we analyzed, our study improves upon them in the following aspects. Firstly, most of previous meta-analyses were performed to assess cancer risk [4],[21],[22],[40], irrespective of the tissue origin. This may affect the reliability of the results due to the irrevocable heterogeneity brought about by different types of cancer. Secondly, the only study with hepatocellular carcinoma and miR-146a [23] failed to detect the association between SNP and HCC risk. This may be partly due to the inability to identify all the studies eligible for analysis. Post publication of the study by Wang *et al*. in 2010 [29], two well-designed studies using large sample size of more ethnic groups were performed by Li *et al*. [30] and Kim *et al*. [20], to further clarify the association. Interestingly, two recently published reports on this topic show similar results. Zhihua Yin’s [41] study found that the miR-146a C variant is associated with a decrease in HCC risk. Yumin Xu’s [42] study suggests that the effect may be enhanced, especially among Asian males. Our meta-analysis was specific to HCC and by far contains the largest number of studies and observations. The reliability of the results by Zhihua Yin [41] could have been affected by the irrevocable heterogeneity brought about by different types of cancer reviewed. This study did not identify the sources of heterogeneity and did not simultaneously sub-group the disease and population. Yumin Xu’s [42] report in HCC also failed to identify all the eligible studies (Wang *et al*. [29] in 2010) and thus excluded almost 2000 samples. Additionally, the report did not describe the model used to combine the ORs. With regard to miR-499 rs3746444, 4 studies [37],[41],[43],[44] showing no significant associations between this SNP and overall cancer risk may have been limited by the heterogeneity introduced by varying types of tissue. Yumin Xu’s [42] study, specifically focused on HCC, also failed to find any significant correlation in all the genetic models. We avoided the heterogeneity brought about by different types of population; thus, we may have detected significantly different vulnerability between CC and TT genotypes in the Chinese population.

### Limitations

Several limitations of this research should be considered when interpreting the results due to some limitations of this meta-analysis. Firstly, our literature searching was depended on English and Chinese databases only. Language bias may exist. Secondly, our study only included three populations (Chinese, Korean and Turkish), so the result may not be able to extrapolation to other populations such as African. Thirdly, lack of available data of many environmental factors including age, HBV/HCV infection status, gender and alcohol consumption may limited the possibility of a more comprehensive evaluation of the association between the SNP and susceptibility to HCC. Fourthly, no significant association was found between miR-499 rs3746444 and HCC under every models may due to the relatively small sample size of those four studies, and should be interpreted with caution. Lastly, even to our best knowledge, we can’t discover all grey literatures.

## Conclusions

We performed comprehensive literature search in multiple databases without limiting publication language and date. We conducted quantitative data synthesis in allele frequency, additive model, dominant model and recessive model. Subgroup analysis was also performed by different characteristics among studies like region distribution, genotyping method and sample size under the additive models. Q-test in all subgroups showed no significant heterogeneity. Our meta-analysis, though with limitations, concludes that there is a strong significant association between rs2910164 in miR-146a and susceptibility to HCC, especially in Chinese population. No significant association was found between miR-499 rs3746444 and HCC under every models, but subgroup study shows subjects with CC genotype in Chinese group are more vulnerable to HCC (OR = 2.171, 95% CI = 1.149-4.104, P = 0.017) than those with TT genotype. This result should be interpreted with caution. Well-designed studies with more ethnic groups are required to further validate the results.

## Competing interests

The authors declare that they have no competing interests that are directly relevant to the content of this study.

## Authors’ contributions

JL was in charge of conceived and designed the study. JL and BX were responsible for collection of data and performing the statistical analysis and manuscript preparation. FJ and SC were responsible for checking the data. WM participated in study design and critically revised the manuscript. All authors were responsible for drafting the manuscript, read and approved the final version.

## Additional file

## Supplementary Material

Additional file 1: Moose checklist. Click here for file
