# Supplementary material for: Association study of two inflammation-related polymorphisms with susceptibility to hepatocellular carcinoma: a meta-analysis
Source: BMC Med Genet. 2014 Aug 10;15:92. doi: 10.1186/s12881-014-0092-7 (PMC4236519; doi:10.1186/s12881-014-0092-7)
Supplement: Additional file 1: — Moose checklist. [file s12881-014-0092-7-S1.pdf]

Generated by Foxit PDF Creator © Foxit Software
http://www.foxitsoftware.com For evaluation only.

MOOSE Checklist

Criteria	Description of the criteria in our meta-analysis	status
	**Reporting of background should include**	
Problem definition	Recent study indicates inflammation and abnormal immune may promotes malignant progression in the development of cancers. Thus makes the inflammation related polymorphisms miR-146 rs2910164 and miR-499 rs3746444 crucial in this process. However, existed studies remain inconclusive and inconsistent	√
Hypothesis statement	The two inflammation related polymorphisms rs2910164 and rs3746444 may associated with HCC.	√
Description of study outcomes	Observed objects developed hepatocellular carcinoma	√
Type of exposure or intervention used	rs2910164 C>G and rs3746444 T>C polymorphisms under allele frequency comparison (C versus G, T versus C); additive model (rs2910164: GC versus GG, CC versus GG, rs3746444:TC versus CC, TT versus CC); dominant model (GC/CC versus GG, TC/TT versus CC); and recessive model (CC versus GC/GG, TT versus CT/CC)	√
Type of study designs used	Case-control studies.	√
Study population	Voluntarily joined with informed consents	√
	**Reporting of search strategy should include**	
Qualifications of searchers	Investigators included experts in genetic epidemiology, biologists, healthcare professional and qualified graduate students. All of the investigators have received training in literature research, statistics and evidence-based medicine.	√
Search strategy, including time period included in the synthesis and keywords	A computer-based search of the most authority database, including PubMed, EMBASE, Web of Science, Cochrane Central Register of Controlled Trials, China National Knowledge Infrastructure (CNKI), China Biological Medicine Database (CBMD) and VIP(Chinese) up to February, 2013. Key words: miR-146a, microRNA, rs2910164, miR-499, rs3746444, polymorphism, Hepatocellular Carcinoma, liver cancer, HCC, Liver neoplasm	√
Databases and registries searched	PubMed, EMBASE, Web of Science, Cochrane Central Register of Controlled Trials, China National Knowledge Infrastructure (CNKI), China Biological Medicine Database (CBMD) and VIP(Chinese)	√
Search software used, name and version, including special features	NoteExpress2 was used to search literature, retrieve citations and eliminate duplications	√
Use of hand searching	Reference lists of obtained articles were searched manually	√

Generated by Foxit PDF Creator © Foxit Software
http://www.foxitsoftware.com For evaluation only.

List of citations located and those excluded, including justifications	The process of literature search and selection are demonstrated in the flow diagram (Figure 1). The reasons for exclusion also given.	√
Method of addressing articles published in languages other than English	Language was not restricted. However few Chinese database was included.	√
Method of handling abstracts and unpublished studies	First, search data base for related studies that may contain same data. Second, contact authors for unpublished data via e-mail. If concrete data was not provided, the study would be excluded.	√
Description of any contact with authors	We contacted two researchers of two studies via e-mail. One of them was unable to find the data, while the other one didn't respond.	√
**Reporting of methods should include**		
Description of relevance or appropriateness of studies assembled for assessing the hypothesis to be tested	We concluded an inclusion and exclusion criteria before searching and was detailed in method.	√
Rationale for the selection and coding of data	The data selected and extracted based on biology relating to the subject including author, year, population, genotyping methods, HWE status, outcome. The data was mainly qualitative variables.	√
Assessment of confounding	Subgroup analysis stratified by study characteristics like population (Chinese and Other), genotyping method (PCR-RFLP and Other), sample size, significant association was performed for a better understanding of the relationship. Sensitive analysis was performed to evaluate the reliability of the meta-analysis.	√
Assessment of study quality, including blinding of quality assessors; stratification or regression on possible predictors of study results	The literature searching and data extraction was performed individually by two investigators. Subgroup analysis stratified by study characteristics like population (Chinese and Other), genotyping method (PCR-RFLP and Other), sample size, significant association was performed for a better understanding of the relationship. Sensitivity analysis to evaluate the reliability of the meta-analysis.	√
Assessment of heterogeneity	Heterogeneity assumption was checked by the chi-square-based Q-test, and a $P > 0.05$ indicates a lack of heterogeneity among studies.	√
Description of statistical methods in sufficient detail to be replicated	Detailed index and stastistical methods are reported in method section.	√
Provision of appropriate tables and graphics	Flow chart to explain searching and selection studies, tables for describe the result and studies characteristics, forest plots for pooled OR, graphs to demonstrate sensitivity analysis.	√
**Reporting of results should include**		

Generated by Foxit PDF Creator © Foxit Software
http://www.foxitsoftware.com For evaluation only.

Graph summarizing individual study estimates and overall estimate	Forest plots are provided individual forest plot. Tables were used for description of the study estimates and studies characteristics.	√
Table giving descriptive information for each study included	Descriptive information for studies of each SNP seperately provided in Table 1/2.	√
Results of sensitivity testing	The results of sensitivity analysis were described in Table 7 and demonstrated in Figure 4.	√
Indication of statistical uncertainty of findings	All the statistical results are uncertain, because statistical significance inevitably contains the possibility to make the type Ⅰ error.	√
**Reporting of discussion should include**		
Quantitative assessment of bias	An asymmetric plot suggests a possible publication bias and the P <0.05 of Egger's test was considered representative of statistically significant publication bias. None was detected under every model.	√
Justification for exclusion	Described in criteria for study selection	√
Assessment of quality of included studies	We thoroughly observed every study and discussed the quality of each included study. Only included the well-designed case-control study that meets our criterias.	√
**Reporting of conclusions should include**		
Consideration of alternative explanations for observed results	The result of miR-499 rs3746444 and HCC fail to testify the hypothesis we proposed. Limitations of the meta-analysis should be considered. Some unknown confounders may alter the result.	√
Generalization of the conclusions	Our meta-analysis, though with limitations, concludes that rs2910164 in miR-146a may confer susceptibility to HCC, especially in Chinese population. No significant association was found between miR-499 rs3746444 and HCC under every models, but subgroup study shows subjects with CC genotype are more vulnerable to HCC than TT genotype in Chinese population.	√
Guidelines for future research	The inflammation related miRNA polymorphisms especially miR-146a plays an important role in immune response and may enlighten our further study in other autoimmune diseases. Further well-designed studies with larger sample size and more ethnic groups are needed to further validate the association between miR-499 and HCC.	√
Disclosure of funding source	None declared conflict of interests. This study does not received financial support of any kind from any foundations.	√
